# Efficacy of Antioxidant Treatment in Reducing Resistin Serum Levels: A Randomized Study

**DOI:** 10.1371/journal.pctr.0020017

**Published:** 2007-05-04

**Authors:** Simona Bo, Giovannino Ciccone, Marilena Durazzo, Roberto Gambino, Paola Massarenti, Ileana Baldi, Antonela Lezo, Elisa Tiozzo, Daniela Pauletto, Maurizio Cassader, Gianfranco Pagano

**Affiliations:** 1 Department of Internal Medicine, University of Turin, Turin, Italy; 2 Unit of Cancer Epidemiology, University of Turin and Centro di Riferimento per l'Epidemiologia e la Prevenzione Oncologica in Piemonte, Turin, Italy; 3 Laboratory of Clinical Nutrition, San Giovanni Battista Hospital, Turin, Italy

## Abstract

**Objectives:**

Few in vitro studies have examined the participation of resistin, a recently discovered adipokine, in oxidative processes. We investigated whether in vivo treatment with the antioxidant vitamin C might affect resistin serum levels.

**Design:**

Randomized prospective open trial.

**Setting:**

San Giovanni Battista Hospital, Turin, Italy.

**Participants:**

Eighty healthy individuals.

**Intervention:**

Administration of 2 g of ascorbic acid orally for 2 wk (*n =* 40; experimental group) or no supplementation (*n =* 40; control group).

**Outcome measures:**

The primary end point was the between-group difference in the before–after change in resistin serum level after vitamin C supplementation. Secondary endpoints were the within- and between-group changes in glucose, insulin, lipid parameters, C-reactive protein fasting values, and markers of oxidative stress.

**Results:**

In the experimental group, vitamin C supplementation was significantly associated with both resistin concentration reduction (from 4.3 ± 1.5 to 2.9 ± 0.8 ng/ml; 95% confidence interval [CI] −1.87, −1.03) and ascorbic acid level increase (from 9.4 ± 2.9 to 19.0 ± 5.2 mg/l; 95% CI 7.9, 11.2). In the control group, resistin levels did not change significantly (from 4.2 ± 1.0 to 4.3 ± 0.9 ng/ml; 95% CI −0.07, 0.37). The between-group differences were highly significant (*p* < 0.001). Vitamin C supplementation was also associated with a statistically significant reduction in nitrotyrosine level and incremental increase in reduced glutathione. In a linear regression model, within-individual changes in vitamin C concentrations were inversely correlated with changes in resistin levels in both groups (each unit increase of vitamin C corresponded to a decrease of about 0.10 units of resistin levels (95% CI 0.13, 0.08; *p* < 0.001).

**Conclusion:**

This is to our knowledge the first randomized trial in humans that has demonstrated that short-term vitamin C supplementation could significantly reduce resistin levels, independent of changes in inflammatory or metabolic variables. Future investigations of resistin participation in oxidative processes are warranted.

## Editorial Commentary

**Background:** Resistin is a hormone that is produced by fat cells. Much of the work on resistin has been done in mice, and as a result of this research the hormone was thought to explain the link between obesity and development of diabetes. In obese mice, higher levels of resistin are seen, and this hormone seems to interfere with the normal role of insulin in reducing blood sugar levels. However, the exact biochemical pathways in mice and humans seem to be very different, and it is not obvious whether resistin plays the same role in the development of diabetes in humans as it does in mice. At the same time, some researchers have suggested links between resistin and oxidative stress, which is thought to be involved in the development of certain diseases, particularly cardiovascular disease. The researchers here wanted to more fully explore these links by finding out whether an antioxidant, vitamin C, affected levels of resistin in blood. The researchers carried out a trial in healthy human participants, who were randomized to receive 2 g of vitamin C daily for two weeks, or no treatment. The primary outcome of the trial was the change in resistin levels in blood, and the researchers also looked at the levels of other biochemical variables in blood, such as fasting glucose, insulin, cholesterol, fatty acids, and nitrotyrosine.

**What the trial shows:** The researchers recruited 80 participants into the trial, and 40 were randomized to receive 2 g of vitamin C supplementation for two weeks. Forty individuals acted as “controls” and received no intervention over the two weeks of the trial. Outcomes were assessed for all but two individuals in the control group. Overall, levels of resistin in blood fell substantially over the course of the trial among the individuals in the vitamin C supplementation group, but not in the control arm of the trial, and this difference between groups was statistically significant. The levels of many other biochemical markers in blood, such as glucose, cholesterol, fatty acids, and insulin, did not show statistically significant changes between the randomized groups. However, levels of two markers of oxidative stress did change: levels of nitrotyrosine, which is associated with cell damage and inflammation, seemed to drop in the vitamin C group relative to the control group, and levels of reduced glutathione (an antioxidant) seemed to increase in the vitamin C group relative to the control group.

**Strengths and limitations:** In this trial, all individuals were randomized at once to the two study groups. While this is unconventional (normally, participants are randomized one by one, as they are screened and deemed eligible for a study), the process would be likely to prevent bias in allocation of individuals to the study groups. Although participants were not blinded to which study group they were assigned to, the laboratory staff measuring biochemical marker levels in blood were blinded to the study groups. A key limitation of this study is that the participants in the control arm did not receive placebo tablets, but rather received no treatment. A placebo control group would have enabled the researchers to blind participants as to whether they received vitamin C or no active intervention. Participants' knowledge of their group assignment (e.g., to receive vitamin C or no intervention) may have affected their response in the trial. Finally, the trial was conducted on a small group of healthy individuals, and no clinical outcomes were examined. Therefore, although the findings are intriguing, their clinical meaning is not clear.

**Contribution to the evidence:** There are few other studies that have been carried out in humans examining the possibility of a link between resistin levels and oxidative stress. This study suggests that vitamin C administration reduces blood levels of resistin in humans. This finding does not yet clearly point to a specific role for resistin in disease processes or human disease, but raises questions for further study.

## INTRODUCTION

Resistin, a recently described adipokine belonging to the cysteine-rich secretory protein family, was originally described as an adipocyte-derived polypeptide that links obesity and insulin resistance in mice [1]. However, in humans, resistin is expressed at very low concentrations in adipose cells [2], but at high levels in mononuclear leukocytes, macrophages, spleen cells, and bone marrow cells [3]. Striking differences in the genomic organization and cellular source of resistin in rodents versus humans make the biological effects found in the mouse not readily transferable to humans [4–6]. Accordingly, an increasing number of reports have raised doubts regarding the possibility of an important relationship between human resistin and various metabolic disturbances characteristic of obesity and type 2 diabetes [7–13].

Resistin was originally found to be related to proteins induced during lung inflammation [14], and the likelihood that it may be involved in the inflammation process [4,12,15–22] is suggested by the high expression levels of resistin in leukocytes, the associations between this protein and inflammatory markers, and the resistin's ability to stimulate in vivo inflammatory cytokines.

Data supporting resistin participation in oxidative processes have been sporadically published. Significant interaction between a single nucleotide polymorphism in the promoter of the human resistin gene and a marker of oxidative stress (NAD(P)H:quinone oxidoreductase I) has been found [23]. Retinoic acid, the acid form of vitamin A, inhibited the expression of resistin in mice and reduced the higher resistin levels of ten men affected by psoriasis [24,25]. Resistin serum levels were inversely associated with nitrotyrosine (NT), a product of free radical activity [22]; resistin inhibited endothelial nitric oxide synthase activation in vitro, thus reducing nitric oxide (NO) bio-availability [26].

The aim of this explicative trial was to evaluate whether an in vivo short-term treatment with an antioxidant vitamin (vitamin C) might substantially affect resistin serum levels. For this purpose, serum resistin values were evaluated in a group of healthy participants, randomized to receive orally 2 g of ascorbic acid daily for 2 wk. Values of fasting glucose, insulin, total and high-density lipoprotein (HDL) cholesterol, triglycerides, and C-reactive protein (CRP) and markers of oxidative stress were measured.

## METHODS

### Participants

After obtaining approval from the San Giovanni Battista Hospital Ethical Committee and informed written consent from participants, a randomized prospective open trial was carried out. Healthy European-descent volunteers aged 20–50 y were recruited from the staff of the San Giovanni Battista Hospital in Turin. Exclusion criteria were as follows: current pregnancy; hyperglycemia (fasting glucose > 6.1 mmol/l); hypertension (blood pressure ≥ 140/90 mm Hg); impaired renal function (serum creatinin ≥ 106 μmol/l); known cardiovascular disease, liver disease, or any other systemic conditions; use of any drug (estrogen included); and being on a particular diet and/or vitamin or other nutrient supplements. All procedures conformed to the principles of the Declaration of Helsinki.

### Outcomes

The primary end point was the between-group difference in the before–after change in resistin serum levels after 2 wk of vitamin C treatment in the experimental group. Secondary end points were the within- and between-group comparisons of changes in the other inflammatory and oxidative variables measured.

### Sample Size

On the basis of our previous data [22], a sample of at least 34 individuals per group was required to detect a standardized difference of 0.5 standard deviations in resistin levels between groups, with a statistical power of 80% and a two-tailed 0.05 α-value. Taking into account the limitations of these assumptions and the possibility of missing some individuals, the total sample size was increased to 40 individuals per group.

### Recruitment

In June–July 2005, a total of 80 eligible participants were enrolled and submitted in the morning, at fasting, to measurements of weight, waist circumference, and blood pressure, and to determinations of serum glucose, total cholesterol, HDL cholesterol, triglyceride, insulin, high-sensitivity CRP (hs-CRP), vitamin A, vitamin C, vitamin E, oxidized and reduced glutathione, NT, and resistin levels. None of the participants showed impaired values of blood pressure, glucose, or cholesterol. Data were collected on smoking habits and physical activity via an interview, and on mean daily nutrient intake via a 3-d food record.

Interventions in the two arms were either daily 2 g of ascorbic acid supplementation or no supplementation for 14 d. Tablets of 1 g of ascorbic acid were consumed twice a day, one at fasting upon awakening and the second 12 h later, every day for 14 consecutive days. All the participants were advised to continue their habitual diet and perform their usual physical activity.

The second blood sample, likewise after 8–12 h of fasting, was collected from the two groups in September–November 2005. The date of the second assessment was fixed in advanced for all participants, well before treatment began for the experimental group, so that any influence of the condition of the participant or his eating patterns on the date of assessment can be ruled out.

In the experimental group a blood sample was collected the morning after the last vitamin C tablet had been taken. During the time lag between the first (June–July 2005) and the second assessment (September–November 2005) no change in health status occurred; participants maintained their weight, waist circumference, and blood pressure values. None of the participants started any treatment (including anti-inflammatory drugs) or were affected by any pathological conditions, including acute or chronic inflammatory conditions or infections.

### Randomization: Sequence Generation

Participants were stratified according to age, sex, smoking habits, body mass index, and fasting levels of glucose, hs-CRP, vitamin C, and resistin. After collection of all baseline data for all participants, the randomization procedure was automatically performed by a statistician, using a SAS program developed to minimize the differences between the two groups for all the stratifying variables. The final distribution was 40 participants on vitamin C supplementation (experimental group) and 40 not supplemented (control group).

### Randomization: Allocation Concealment

When the results of baseline tests were available for all 80 participants, random allocation with a minimization algorithm was centrally performed in a single step. The researchers received then two lists of nominative data (40 for the experimental group and 40 for the control group). In this way the possibility for researchers to predict or influence the allocation of participants was completely prevented.

### Measurements

The 3-d food record consisted of a detailed written food diary kept prospectively: the participants were instructed to record everything they ate or drank during two consecutive week days and one weekend day.

Weight and waist and hip circumference were measured using standard protocols. Systolic and diastolic blood pressures were measured twice with a standard mercury sphygmomanometer in a sitting position, after at least 10 min of rest. Values reported are the mean of the two determinations.

Physical activity during leisure time was defined as light (inactive, <4 h/wk), moderate (4 h/wk), or heavy (>4 h/wk) [27].

Serum glucose was measured by the glucose oxidase method, and triglycerides and HDL cholesterol by enzymatic colorimetric assay (Hitachi 911 Analyzer, Sentinel, Milan, Italy). Serum insulin values were determined by immunoradiometric assay (Radim, Pomezia, Italy; intra-assay coefficient of variation (CV) 1.9%, inter-assay CV 6.2%). Serum CRP levels were measured by a high-sensitivity latex agglutination method (hs-CRP) on the Hitachi 911 Analyzer (intra-assay and inter-assay CVs respectively 0.95% and 1.3%). Serum NT values were determined by an ELISA kit (HyCult Biotechnology Pantec, Turin, Italy; inter-assay precision 3.3%; intra-assay CV 5%). Serum resistin values were analyzed by ELISA (BioVendor, Brno, Czech Republic); the intra-assay and inter-assay CVs were, respectively, 2.8% and 5.5%.

Plasma vitamin C levels were detected by HPLC (Solvent Delivery System 9012, with a UV-Visible Detector 9050, Varian, Walnut Creek, California, United States); intra-assay and inter-assay CVs respectively 4.8% and 5.9%). Plasma vitamin A and vitamin E were simultaneously evaluated by HPLC (Chromsystems Instruments, Munich, Germany; intra-assay and inter-assay CVs respectively 3.3% and 5.6%). Total and reduced glutathione in red blood cells were analyzed by HPLC after a derivatization of hemolyzed samples with ammonium 7-fluorobenzo-2-oxa-1,3-diazole-4-sulphonate, using a fluorescence detector (Varian 9070) with excitation at 385 nm and emission at 515 nm. Oxidized glutathione was calculated as the difference between total and reduced glutathione (intra-assay and inter-assay CVs respectively 4.1% and 5.3%).

All blood samples underwent laboratory testing in blind.

### Statistical Methods

We applied the Student's *t-*test for paired data to investigate before–after changes in the concentrations of several blood variables within the experimental and control groups. To assess whether these before–after changes were different between the two groups, *t*-tests for independent samples, assuming either equal or unequal variances, were performed.

In order to compare the between-group differences of variables with different units of measurement, all the comparisons were done using the corresponding standardized values (mean/overall standard deviation).

Since the distributions of hs-CRP, insulin, and triglyceride values were positively skewed, their levels were log-transformed, in order to approximate a normal distribution. In all the statistical analyses the log-transformed values of these variables were used, but for descriptive purposes, median (and interquartile range) of untransformed values are reported.

To explore the biological relationship between resistin and vitamin C, we fitted a linear regression model, using before–after change of resistin as the response variable and change of ascorbic acid level and group (experimental or control) as predictors. To reduce the influence of outliers, a robust linear regression technique, using Huber and Tukey bi-weights, was chosen [28,29]. Since neither the group variable nor the interaction between group and vitamin C change were statistically significant when the effect of vitamin C change was accounted for, only the latter was retained in the model as an explicatory variable.

All the statistical analyses were performed with STATA (v. 8.0; StataCorp, College Station, Texas, United States).

## RESULTS

### Participant Flow

**Figure 1:**
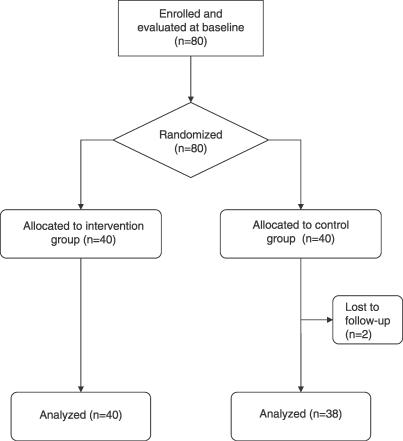
Flow Diagram of the Trial

Out of the 40 participants in the control group, two were lost during follow-up (they moved away). No participant discontinued treatment or was lost during follow-up in the experimental group. Data from 78 participants were thus analyzed. The flow diagram of the trial is reported in Figure 1.

### Baseline Data

Basal clinical and laboratory characteristics of all the enrolled participants by group are shown in Table 1. No meaningful difference was evident between the two groups. Habitual nutrient intake patterns were very similar between the two groups; in particular, estimated vitamin C intake from food was 142.5 ± 77.2 and 144.8 ± 70.4 mg/d, respectively, in the experimental and control groups. Similarly, intake of total calories; total fat; polyunsaturated, saturated, and monounsaturated fat (as energy percent); fiber; and vitamin A and E were not significantly different between the two groups (data not shown).

**Table 1:**
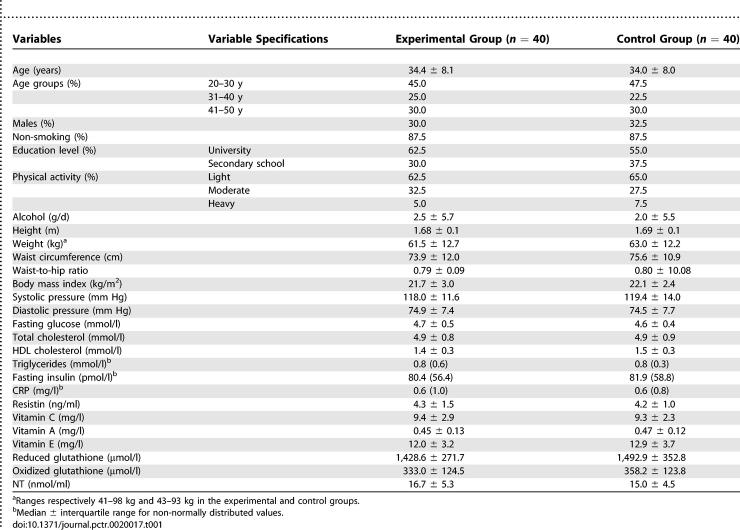
Clinical and Laboratory Characteristics of the Participants Studied

### Outcomes and Estimation

After vitamin C supplementation, ascorbic acid plasma levels significantly increased in the experimental group, while a slight reduction was observed in the control group (Table 2). Resistin serum concentrations showed a significant reduction in the experimental group (from 4.3 to 2.9 ng/ml) and a small, not significant, increase in the control group (from 4.2 to 4.3 ng/ml). The differences between groups in the blood levels of vitamin C and resistin after supplementation were highly significant (*p* < 0.001) (Table 2). Reduced glutathione concentrations increased in both groups, but the increment was more than 2-fold higher in the experimental group. Levels of NT significantly decreased in the experimental group, and slightly increased in the controls, giving a between-group difference of borderline statistical significance. Values of hs-CRP slightly increased in the controls, and no significant change was evident for fasting glucose, insulin, lipid variables, or other antioxidant vitamins within or between groups.

**Table 2:**
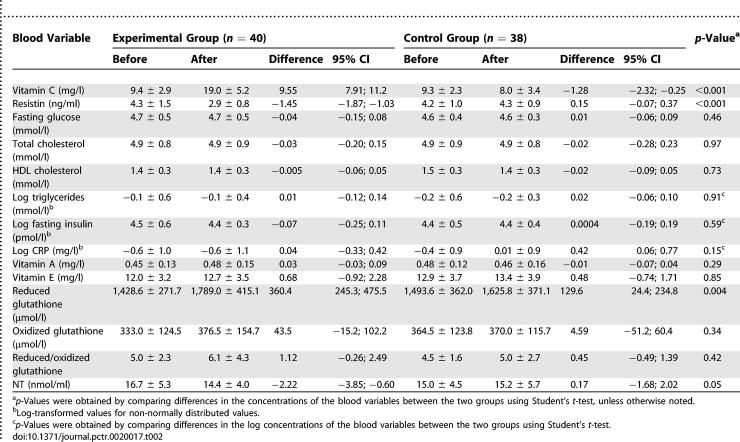
Concentrations of Different Blood Variables before and after Vitamin C Supplementation by Group: Absolute Difference (End-of-Study Minus Baseline Values) with 95% CIs

**Figure 2:**
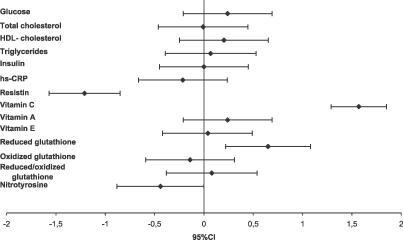
Means and 95% CIs of Between-Group Differences of Changes (in Standardized Units) for the Blood Variables Compared (Experimental Minus Control Group) For this analysis, all the variables were standardized (mean/overall standard deviation).

Figure 2 shows the standardized between-group differences (and 95% CI) for the evaluated blood variables. Apart from the large differences recorded for vitamin C and resistin concentrations, only NT and reduced glutathione variations were affected by the antioxidant treatment.

According to the results of the robust linear regression, changes in resistin serum levels were inversely related to changes in vitamin C plasma levels both in the experimental and control groups. On the whole sample, a one-unit increase in before–after change of vitamin C corresponded to a statistically significant decrease (beta = −0.10; 95% CI −0.13, −0.08; *p* < 0.001) in the mean change of resistin. This overall relationship still holds after adjusting by group (beta = −0.08; 95% CI −0.13, −0.03; *p* = 0.003).

**Figure 3:**
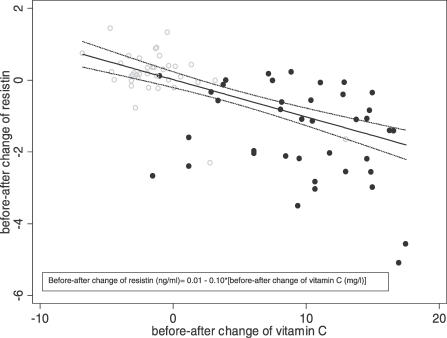
Before–After Change in Resistin versus Before–After Change of Vitamin C Overall plot of observed (circles) and predicted values (solid line) of variation of resistin (with 95% confidence bands; dotted lines) according to the before–after change of vitamin C concentrations (end-of-study minus baseline values). Solid black circles represent the experimental group; open grey circles represent the control group.

Figure 3 reports the observed and predicted values, with 95% confidence bands, estimated for both experimental and control groups.

### Adverse Events

Two participants in the experimental group complained of gastric discomfort after vitamin C supplementation.

## DISCUSSION

### Interpretation

The results of this trial clearly indicated that in healthy individuals resistin serum levels were significantly reduced by a short period of supplementation with vitamin C. This treatment was also associated with other changes in oxidation-related variables (reduction of NT levels and incremental increase of reduced glutathione).

To date, there has been considerable controversy surrounding the physiological relevance of resistin: the associations with insulin resistance and obesity, reported in mice, have not been conclusively demonstrated in humans [7–13]. Growing evidence has suggested that this protein, highly expressed in monocytes and macrophages in humans, might have an involvement in the inflammation process, either being induced by increased cytokine levels, or directly stimulating the production of pro-inflammatory cytokines, thus leading to inflammation amplification [4,12–22]. Recent studies found that resistin activated human endothelial cells in vitro, inducing the expression of endothelin 1, adhesion molecules, and chemokines [16,30], and stimulated smooth muscle cell proliferation [31]. Endothelial dysfunction and/or pro-inflammatory effects could represent, thus, the link between higher resistin serum levels and increased prevalence of cardiovascular diseases in humans. This latter association was demonstrated by some studies [20,32,33] and supported by the finding of elevated resistin secretion from macrophages infiltrating atherosclerotic arterial walls [34].

It was recently demonstrated that resistin impairs endothelium-dependent vasorelaxation in porcine coronary arteries, by reducing NO bio-availability [35]. Possible participation of resistin in oxidative processes has been sporadically suggested [22–26], and an impact of oxidative stress on the regulation of murine adipokine gene expression was proposed [36]. The possibility that antioxidants might act on resistin expression and levels has been suggested by a few studies on animals: retinoic acid inhibits the expression of resistin in mice [24], and the antioxidant seleno-methionine, which increases the activity of glutathione peroxidase in endothelial cells, completely blocks vasomotor dysfunction induced by resistin in porcine arteries [35]. In humans, a small study testing the effect of a chronic treatment with retinoid therapy on patients with psoriasis showed normalization of the increased resistin serum levels, together with a reduction in insulin sensitivity, without any changes in circulating adiponectin or tumor necrosis factor concentrations [25].

Our data clearly show that the antioxidant supplementation significantly reduced resistin serum levels, but changed neither metabolic parameters nor hs-CRP levels (Table 2), the latter being, however, already very low at baseline. In the control group, unexpectedly, we observed a slight, statistically significant reduction in vitamin C plasma levels and a small increase in serum resistin values (Figure 3). This relationship was independent from changes in other metabolic/inflammatory parameters, in line with the absence of correlations between tumor necrosis factor and resistin changes observed in males with psoriasis who received retinoid treatment [25]. This suggested a specific direct effect of antioxidant substances on resistin expression from monocytes/macrophages. Accordingly, the reduction in resistin levels was maintained 3 mo after retinoid treatment in males with psoriasis, unlike the transient effect found by the authors on insulin sensitivity [25].

It has been suggested that resistin inhibits endothelial nitric oxide synthase activation in vitro and increases superoxide radical production in porcine coronary arteries [35] and human endothelial cells [26], thus reducing NO bio-availability. Imbalance in the production and regulation of oxygen radicals and the subsequent oxidative inactivation of NO leads to oxidative stress and might contribute to vascular disease [36].

The reaction of NO with superoxide anion radicals (O_2_^•−^) yields peroxynitrite (ONOO^−^), which can oxidize many bio-molecules. NT, generated from the oxidation of tyrosine, has been considered as a measure of ONOO^−^ oxidative injury, and elevated plasma levels have been reported in conditions associated with oxidative stress, such as diabetes [37–39]. In the experimental group, NT levels were significantly reduced after the supplementation, while a very slight increase was evident in the control group. These changes might be due to the direct protective antioxidant effect of vitamin C. Ascorbic acid could stimulate reduced glutathione synthesis, and, thus, might be responsible for the statistically significant incremental increase in its levels found in the experimental group [40].

Our study is to our knowledge the first human randomized trial carried out on a very homogeneous sample of healthy volunteers that has demonstrated that short-term supplementation with an antioxidant vitamin might significantly reduce resistin serum levels, independent of changes in inflammatory and metabolic variables. This finding, together with in vitro results [26,35], indicates that oxidative stress may be one of the major mechanisms by which resistin acts and is regulated. It could be hypothesized that the raised resistin values occasionally found to be associated with type 2 diabetes or obesity and, more consistently, with endothelial dysfunction and inflammation, rather than being linked to insulin resistance, might be associated with increased oxidative stress, common to all these conditions [41,42].

### Limitations

Oxidative stress is not an easily definable condition, and none of the indices used for its evaluation could be defined as the most appropriate criterion in universal terms [43]. Problems of low protocol adherence were not documented. Contamination between the two groups seems unlikely due to the short-term and simple regimen of the intervention.

The open nature of the study and the lack of a placebo might have affected participants' behavior during the trial. However, it is quite unlikely that this could represent a source of measurement bias, since all the end points (e.g., the relationship between vitamin C and resistin levels) were blood measurements, blindly assessed by the same laboratory.

It is unlikely that dietary patterns would have changed substantially from June–July 2005 to September–November 2005; moreover, all the participants were advised to continue their habitual diet. As participants were randomly allocated in the two groups and were similar for many environmental characteristics (smoking, alcohol and nutrient intake, education level, and physical activity), there is no reason why only one group should have changed dietary habits. Vitamin C levels slightly decreased in the control group, and a seasonal effect might be the most probable cause (higher fruit/vegetable intake is expected during the summer). In the experimental group, this change might not be appreciable, owing to the ascorbic acid supplementation received. The slight reduction in vitamin C plasma levels of the control individuals corresponded to a small increase in their serum resistin values. This unexpected finding is not only consistent with but it also further strengthens the results found in the experimental group.

The loss of two volunteers in the control group, because they moved away, is unlikely to have influenced the main results of the study. An intention-to-treat analysis, assuming for these two control individuals the same average resistin reduction of the experimental group, confirmed the results.

Until further studies confirm these results in other cohorts (patients with diabetes, obesity, etc.) and the potential benefits of antioxidant treatment are demonstrated, no clinical consideration could be extrapolated from our data.

### Generalizability

This explicative trial was performed in healthy volunteers, and it may be reasonable to generalize its results to healthy groups. Further studies are needed to test these relationships in individuals with health problems or who are taking medication.

### Overall Evidence

Existing evidence on resistin participation in oxidative processes is scarce and heterogeneous; most studies were performed in vitro or in animals [23,24,26]. In humans, a small study on patients with psoriasis showed normalization of the increased resistin serum levels after treatment with retinoid therapy [25]. This is to our knowledge the first trial in humans designed to assess the impact in vivo of antioxidant treatment on resistin serum levels. Its main result is that vitamin C significantly reduced resistin, without changing other metabolic/inflammatory parameters. This suggests a specific direct effect of antioxidant substances on resistin expression from monocytes/macrophages.

### Conclusion

The exact biological role of resistin in humans remains so far equivocal. These results add some data to currently available knowledge by demonstrating an inverse relationship between blood levels of resistin and vitamin C. Future investigations on resistin participation in oxidative processes are warranted to further clarify the intriguing, but not well-defined role of this protein in chronic degenerative diseases.

## SUPPORTING INFORMATION

CONSORT Checklist (41 KB DOC)

Trial Protocol (47 KB DOC)

## Abbreviations

CI: confidence interval
CRP: C-reactive protein
CV: coefficient of variation
HDL: high-density lipoprotein
hs-CRP: high-sensitivity C-reactive protein
NO: nitric oxide
NT: nitrotyrosine
