# Supplementary material for: Efficacy of Antioxidant Treatment in Reducing Resistin Serum Levels: A Randomized Study
Source: PLoS Clin Trials. 2007 May 4;2(5):e17. doi: 10.1371/journal.pctr.0020017 (PMC1865087; doi:10.1371/journal.pctr.0020017)
Supplement: Trial Protocol — (47 KB DOC) [file pctr.0020017.sd002.doc]

PROTOCOLLO DELLO STUDIO

STUDIO RANDOMIZZATO SULL’EFFETTO DI UN TRATTAMENTO ANTIOSSIDANTE SUI LIVELLI SIERICI DI RESISTINA

STUDY PROTOCOL

EFFECT OF AN ANTI-OXIDANT TREATMENT ON RESISTIN SERUM LEVELS.

# A RANDOMIZED STUDY

###### INTRODUCTION

# 1.1 Background

Resistin, a recently described adipokine, belonging to the cysteine-rich secretory protein family, was originally described as an adipocyte-derived polypeptide, that provides the link between obesity and insulin resistance in mice^1-2^. However, resistin is expressed at very low concentrations in human adipose cells, whereas at high levels in mononuclear leukocytes, macrophages, spleen, bone marrow cells. The striking differences in the genomic organization and cellular source of resistin in rodents make the biological effects found in the mouse not readily transferable to humans^3-5^.

Accordingly, an increasing number of reports raised doubts regarding the possibility of an important relationship between human resistin and various metabolic disturbances, characteristic of obesity and type 2 diabetes^4-5^.

Resistin was originally found to be related to proteins induced during lung inflammation^6^, and the high expression levels of resistin in leukocytes, the associations between this protein and inflammatory markers, the property to stimulate in vivo inflammatory cytokines suggest the possibility that resistin may be involved in the inflammation process^6^.

Data supporting the participation of resistin in oxidative processes have been sporadically published. A significant interaction among a single nucleotide polymorphism in the promoter of the human resistin gene and a marker of oxidative stress (NAD(P)H:quinone oxidoreductase I) has been found^7^. Retinoic acid, the acid form of vitamin A, inhibited the expression of resistin in mice and reduced the higher resistin levels of ten males affected by psoriasis^8^. Resistin serum levels were inversely associated with nitrotyrosine (NT), a product of free radical activity^9^.

A pilot-analysis performed in five healthy volunteers showed that oral administration of vitamin C for 15 days determined a slight reduction in resistin serum levels.

**1.2 Aim**

The aim of the present study is to evaluate whether an in vivo treatment with an antioxidant vitamin (vitamin C) might substantially affect resistin serum levels.

For this purpose, serum resistin values will be evaluated in a group of healthy subjects, randomized to receive orally 2 g ascorbic acid for two weeks. Values of fasting glucose, insulin, total and HDL-cholesterol, triglycerides, C-reactive protein (CRP), and markers of oxidative stress will be measured.

# 2. STUDY DESIGN

Two-arms open prospective randomized study.

# Subjects

# Healthy volunteers aged 20-50 years will be recruited from the staff of the S.Giovanni Battista Hospital of Turin.

# Inclusion criteria are: age 20-50 years, absence of known hyperglycemia, hypertension, cardiovascular disease, impaired renal function, liver disease, or any other systemic conditions, no use of any drug -estrogen included-, not being on a particular diet and/or vitamin or other nutrient supplementation

# Exclusion criteria are: actual pregnancy, known hyperglycemia, hypertension, cardiovascular disease, impaired renal function, liver disease, or any other systemic conditions, use of any drug -estrogen included-, being on a particular diet and/or vitamin or other nutrient supplementation; history of renal calculi.

## 2.2 Randomization

After a full baseline evaluation of all included subjects, they will be stratified according to:

- age,
- sex,
- smoking habits,
- body mass index (BMI),
- fasting blood levels of glucose, hs-CRP, vitamin C, and resistin.

## The randomization procedure will be automatically performed by a statistician, using a SAS program developed to minimize the differences between the two groups for all the stratifying variables.

## 2.3 Treatments

Subjects will be randomly allocated, within each stratum, to receive either daily 2 g ascorbic acid (Cebion® 1000 per 2/die) supplementation or no supplementation for 14 days.

The final distribution will be: 40 subjects on vitamin C supplementation (experimental group) and 40 not supplemented (control group).

These dosages were demonstrated to be safe also for prolonged treatments^10^

**2.4 Endpoints**

Main endpoints:

Mean resistin serum level reduction, after vitamin C treatment, of at least 0.50 standardized units.

Secondary endpoints:

The within- and between-group variations in the other inflammatory and oxidative variables measured.

- 1. **Sample size**

On the basis of our previous data^9^, a sample of at least 34 subjects per group is required to detect a standardized difference of 0.5 DS resistin reduction in the experimental group, with a statistical power of 80% and two-tailed 0.05 α-value. Taking into account the limitations of these assumptions and the possibility of missing some individuals, the total sample size is increased to 40 subjects per group.

**2.6 Statistics**

The *t*-Student test for paired data will be used to investigate variations in the concentrations of several blood variables in the experimental group and in the control group. To assess whether these changes are different between the two groups, *t*-tests for independent samples, assuming either equal or unequal variances, will be performed.

**2.7 Measurements**

All the subjects will be submitted in the morning, at fasting, before and after the treatment, to measurements of weight, waist circumference, blood pressure, and to determinations of serum glucose, total cholesterol, HDL-cholesterol, triglyceride, insulin, high sensitivity C reactive protein (hs-CRP), vitamin A, C, E, oxidized and reduced glutathione, NT, and resistin levels.

Data about smoking habits, physical activity and mean daily nutrient intakes will be collected, respectively, by an interview, and a validated 3-day food record.

**3. REFERENCES**

1. Steppan CM, Bailey ST, Bhat S, et al. The hormone resistin links obesity to diabetes. Nature 2001; 18: 307-312
2. Steppan CM, Brown EJ, Wright CM, et al. A family of tissue-specific resistin-like molecules. Proc Natl Acad Sci USA 2001; 98: 502-506
3. Hotamisligil GS. The irresistible biology of resistin. J Clin Invest 2003; 111: 173-174
4. Savage DB, Sewter CP, Klens ES, et al. Resistin/FIZZ3 expression in relation to obesity and peroxisome proliferator-activated receptor γ action in humans. Diabetes 2001; 50: 2199-2202
5. Janke J, Engeli S, Gorzelniak K, Luft FC, Sharma AM. Resistin gene expression in human adipocytes is not related to insulin resistance. Obes Res 2002; 10: 1-5
6. Gomez-Ambrosi J, Frühbeck G. Do resistin and resistin-like molecules also link obesity to inflammatory diseases? Ann Intern Med 2001; 135: 306-307
7. Smith SR, Bai F, Charbonneau C, Janderova L, Argyropoulos G. A promoter genotype and oxidative stress potentially link resistin to human insulin resistance. Diabetes 2003; 52: 1611-1618
8. Felipe F, Bonet ML, Ribot J, Palou A. Modulation of resistin expression by retinoic acid and vitamin A status. Diabetes 2004; 53: 882-889
9. Bo S, Gambino R, Pagani A, et al. Relationships between human serum resistin, inflammatory markers and insulin resistance. Int J Obes 2005;29:1315-1320
10. Tousoulis D, Antoniades C, Tentolouris C, Tsioufis C, Toutouza P, Stefanadis C. Effects of combined administration of vitamins C and E on reactive hyperemia and inflammatory process in chronic smokers. Atherosclerosis 2003; 170: 261-267.
